# Improved Outcomes of Childhood Acute Lymphoblastic Leukemia: A Retrospective Single Center Study in Saudi Arabia

**DOI:** 10.31557/APJCP.2019.20.11.3391

**Published:** 2019

**Authors:** Abdullateef Mohammed Ahmed, Hassan Al-Trabolsi, Mohammed Bayoumy, Ibraheem Abosoudah, Fawwaz Yassin

**Affiliations:** *King Faisal Specialist Hospital and Research Center, Jeddah Branch, Alrawdah, Jeddah, Makkah, Kingdom of Saudi Arabia. *

**Keywords:** Acute lymphoblastic leukemia, central nervous system, chromosomal abnormalities

## Abstract

**Objective:**

Understanding the clinical and genetic characteristics of pediatric acute lymphoblastic leukemia (ALL) patients may help assigning the appropriate treatment. This study aims to understand patients’ characteristics, “real-world” treatment practice and outcomes of pediatric ALL.

**Methods:**

A cohort of 213 pediatric ALL patients, treated at (King Faisal Specialist Hospital and Research Center –Jeddah branch) KFSH and RC-J during the period of January 2002 to December 2015 were analyzed retrospectively. Statistical analyses were performed on patients’ demographic, clinical and genetics characteristics and outcomes of different treatment protocols. Survival was evaluated using Kaplan-Meier method, and differences in survival were tested using Log-Rank. Significance was set at 0.05 level.

**Results:**

Median age of the study cohort was 5 years (range 0.5–15 years) with 55.4% of male population. Majority of the patients had pre-B-cell ALL (88.7%), WBC count <50, 000/µL at diagnosis (76.1%, median = 13.5/µL with a range of 0.51–553.0/µL) with involvement of central nervous system (CNS) disease in 8.5%patients.Different common chromosomal anomalies or abnormalities, including t(12, 21) translocation, MLL genre arrangements, trisomy (4, 10, 17)and others, were detected. Early response to the risk-directed treatment received by the patients (91.1% achieving <5% blast in the bone marrow) as well as the end of induction outcome (96.2%) was encouraging.

**Conclusion:**

We found that the patients’ clinical characteristics and distribution of genetic abnormalities were similar to those of the western countries. Our findings show that the earlier gap between the western countries and KSA in terms of survival has been closed and that competitive outcomes can be achieved with local infrastructure.

## Introduction

Treatment of childhood acute lymphoblastic leukemia (ALL) relies on risk-based stratification, and patients are classified on the basis of risk of treatment failure. Several clinical characteristics- age, initial white blood cell (WBC) count, involvement of the central nervous system (CNS) disease at diagnosis, involvement of testis at diagnosis, immunophenotype, cytogenetics abnormalities-have been found to be associated with disease prognosis and outcomes. Patients with favorable prognosis are treated with less toxic regimen, while more aggressive regimens are offered to those with poor prognosis. The main components of treatment based on multidrug chemotherapy regimen to avoid drug resistance includes-remission induction, consolidation, interim maintenance, delayed intensification, maintenance chemotherapy, and CNS directed therapy (Cooper and Brown, 2015). With the incremental advances in therapy more than 80% survival (98% in certain subset) has been achieved in the developed countries (Gaynon et al., 1997). However, treatment outcome in the developing countries lags far behind with an average survival rate of 35% mainly due to poor financial status, delay in diagnosis, treatment abandonment, and inadequate supportive care (Al-Nasser et al., 2008 ; Howard et al., 2008; Ribeiro et al., 2008; Liang et al., 2010). 

According to the Saudi Cancer Registry report published in 2013, childhood cancer accounts for 6.1% among all cancer, and acute lymphoblastic leukemia accounts for highest incidence of 31% (Saudi Cancer Registry MoH, 2013). In KSA, different study groups either collaboratively or individually have been working tirelessly to improve “real-world” treatment practice and outcome for childhood ALL. For example, Middle East Childhood Cancer Alliance (MECCA) and the Saudi Arabian Pediatric Hematology/Oncology Society (SAPHOS) have conducted collaborative studies prospectively as well as retrospectively to collect data, which would serve as the basis for the subsequent clinical studies. Many single center/institutional studies have also been conducted to assess patients’ characteristics and outcome of childhood ALL in KSA and to compare survival with respect to developed countries (Al-Nasser et al., 2008; Aur et al., 1985; Khalil et al., 1994). The present study is another addition to the efforts towards improving outcome of childhood ALL in KSA. 

## Materials and Methods

*Patients and Study variables*

This is a retrospective study involving 213 pediatric ALL patients (0.5–15 years), who were treated in KFSH and RC-J branch, (Kingdom of Saudi Arabia) (KSA) during a period of 14 years from January 2002 to December 2015. The study was approved by the Institutional Research Ethics Committee. The informed consent was waived since the data were analyzed retrospectively. The patients’ data were extracted from the hospital’s electronic medical record (EMR)for the study period of 14 years. The data include patients’ demographics, clinical and genetic characteristics (at diagnosis) including WBC count, immunophenotype, cerebrospinal fluid cytology, cytogenetics study, NCI risk group assignment, treatment protocols used (CCG-1882A, CCG-1882B, CCG-1891, CCG-1961RegD, CCG-1961 RegC, CCG 1991/IS, COG0232, ST.J.XIII/BH, and ST.J.XV/BH), early response to therapy (on day14 of induction) and remission status at the end of induction of therapy, occurrence and site of relapse, and the patient’s status at the most recent chart entry.

Bone marrow (BM) evaluation was carried out to assess response to therapy. Out of 213 patients, 207 patients were assessed on Day 14 of induction for early response. All patients who completed induction were assessed for remission status based on the BM morphological evaluation (M1<5% blast, M2 = 5–15%, and M3>15% blast. Of 189 patients with pre B-cell ALL, only 51 patients were assessed for minimal residual disease (MRD) by flowcytometry method in January 2013as the hospital did not have access to the facility until 2013.Out of 213, 11 patients were lost during follow-up while one patient was referred to other center.

*Statistical Analysis*

Evaluation of dichotomous variables was done using Fisher exact test, and the data are presented as percentages and number of cases. Categorical data were analyzed using Chi-square test. Continuously distributed variables were presented as the mean ± standard deviation. Survival was evaluated using Kaplan-Meier analyses and differences in survival were tested using either the Log-Rank. Duration of overall survival (OS) was defined as time from diagnosis until death from any cause; patients remaining alive were censored at the date of last contact. Event-free survival (EFS) was calculated from the date of diagnosis until the occurrence of an event; events were defined as failure to achieve remission at the end of the induction phase of therapy, relapse after achieving a complete remission (CR) and death from any cause. Statistical analyses were performed using the Statistical Package for Social Sciences, version 21.0 (SPSS, Chicago, Ill), and P<0.05 was considered statistically significant.

*Definitions*

Overall survival (OS) was defined as the duration from the diagnosis index date to the end of the study period or until death from any cause; patients remaining alive were censored at the date of last contact. EFS was calculated from the date of diagnosis until the occurrence of an event; events were defined as failure to achieve remission at the end of the induction phase of therapy or as relapse after achieving a complete remission (CR) or death from any cause. 

**Table 1:** Demographics, Clinical, and Laboratory Characteristics of Children with ALL

Features	Total (n=213)	NCI-HR (n=131)	NCI-LR (n=82)
Age			
<10 years	175 (82.2)	95 (44.6)	80 (37.6)
≥ 10 years	38 (17.8)	36 (16.9)	2 (0.9)
Median age (range)	5 (0.5 -15) years	5 **(?**	4 **(?**
Mean age (±SD)	5.5±3.6	6.1±4.1	4.4±2.4
Gender			
Male	118 (55.4)	75 (35.2)	43 (20.2)
Female	95 (44.6)	56 (26.3)	39 (18.3)
Immunophenotype			
Pre-B cell	189 (88.7)	109 (51.2)	80 (37.6)
T-cell	24 (11.3)	22 (10.3)	2 (0.9)
CNS status			
CNS I	182 (85.4)	101 (47.4)	81 (38)
CNS II	13 (6.1)	12 (5.6)	1 (0.6)
CNS III	18 (8.5)	18 (8.5)	0 (0)
WBC (x10^9^)			
<50	162 (76.1)	82 (38.5)	80 (37.6)
≥ 50	51 (23.9)	49 (23)	2 (0.9)
Down’s syndrome			
No	201 (94.4)	123 (57.5)	78 (36.6)
Yes	12 (5.6)	8 (3.8)	4 (1.9)
Chromosomal translocation			
BM-t1221	31 (14.6)	21 (67.7)	10 (33.3)
BM-MLL	5 (2.3)	5 (100)	0 (0)
BM-trisomies (4, 10, 17)	39 (18.3)	23 (58.9)	16 (41.1)
Others	69 (32.4)	52 (75.4)	17 (24.6)
DNA index			
<1.16	62 (29.1)	41 (66.1)	21 (33.9)
≥ 1.16	16 (7.1)	6 (37.5)	10 (62.5)
Undetermined	135 (63.4)		
Cytogenetic studies			
Normal	24 (11.3)	12 (50)	12 (50)
Abnormal	59 (27.7)	39 (66.1)	20 (33.9)
Undetermined	130 (61.0)		
Site of relapse			
BM	25 (11.7)	16 (64)	9 (36)
CNS	5 (2.3)	5 (100)	0 (0)
BM+CNS	1 (0.45)	1 (100)	0 (0)
Testis	1 (0.45)	1 (100)	0 (0)
Relapse	5 (15.6)	21 (65.6)	6 (22.8)
Day 14 of induction			
M1	194 (91.1)	117 (60.3)	77 (39.7)
M2	12 (5.6)	8 (66.6)	4 (33.3)
M3	1 (0.5)	1 (100)	0 (0)
End of induction outcome			
M1	205 (96.2)	126 (61.5)	79 (38.5)
M2	2 (0.9)	2 (100)	0 (0)

**Table 2:** Outcome Estimates of All Patients

	N (%)
Alive	182 (85.4)
Off therapy with remission	176 (82.6)
Alive with disease	6 (2.8)
Dead	31 (14.6)
Disease-related	21 (9.9)
Treatment-related	10 (4.7)
Death at induction	4 (2)
Toxicit y-related	2 (1)
Leukemia progression	2 (1)
Lost follow-up	11 (5.2)
Refereed to other center	1 (0.5)
Total	213

**Table 3:** Association of Different Variables with Outcome in ALL Patients

Variables	N	Remission	Dead	Active	p-value
Age	213				
<10 years		147 (84)	23 (13.1)	5 (2.9)	0.456
≥ 10 years		29 (76.3)	8 (21.1)	1 (2.6)	
Mean age (±SD)					
Gender	213				
Male		90 (76.3)	23 (19.5)	5 (4.2)	0.022
Female		86 (90.5)	8 (8.4)	1 (1.1)	
Immunophenotype	213				
Pre-B cell		161 (85.2)	24 (12.7)	4 (2.1)	0.016
T-cell		15 (65.5)	7 (29.2)	2 (8.3)	
CNS status	213				
CNS I		155 (85.2)	21 (11.5)	6 (3.3)	0.023
CNS II		10 (76.9)	3 (23.1)	0 (0)	
CNS III		11 (61.1)	7 (38.9)	0 (0)	
WBC (x109)	213				
<50		139 (85.8)	19 (11.7)	4 (2.5)	0.09
≥ 50		37 (72.5)	12 (23.5)	2 (3.9)	
Down’s syndrome	213				
No		167 (83.1)	28 (13.9)	6 (3)	0.496
Yes		9 (75)	3 (25)	0 (0)	
Chromosomal translocation					
BM-t1221	31	29 (93.5)	1 (3.2)	1 (3.2)	0.407
BM-MLL	5	2 (40)	2 (40)	1 (20)	<0.001
BM-trisomies (4, 10, 17)	39	38 (97.4)	1 (2.6)	0 (0)	0.082
Others	69	54 (78.3)	11 (15.9)	4 (5.8)	-
DNA index	78				
<1.16		54 (87.1)	8 (12.9)	0 (0)	0.195
≥ 1.16		16 (100)	0 (0)	0 (0)	
Site of relapse	32				
BM		3 (12)	17 (68)	5 (20)	
CNS		1 (20)	3 (60)	1 (20)	0.68
BM+CNS		1 (100)	0 (0)	0 (0)	
Testis		0 (0)	1 (100)	0 (0)	

**Figure 1:**
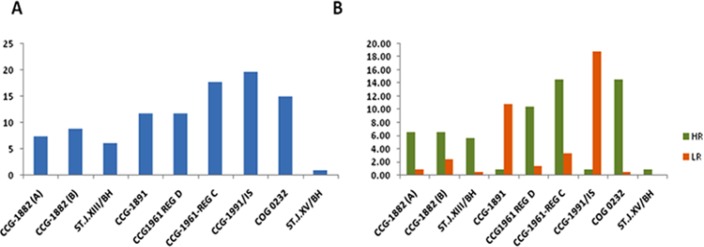
Frequency of Treatment Protocols Used. (A), Protocols frequently used for treating ALL children (p<0.001); (B), Abundance of protocol use for treating high risk and low risk ALL children (p <0.001)

**Figure 2:**
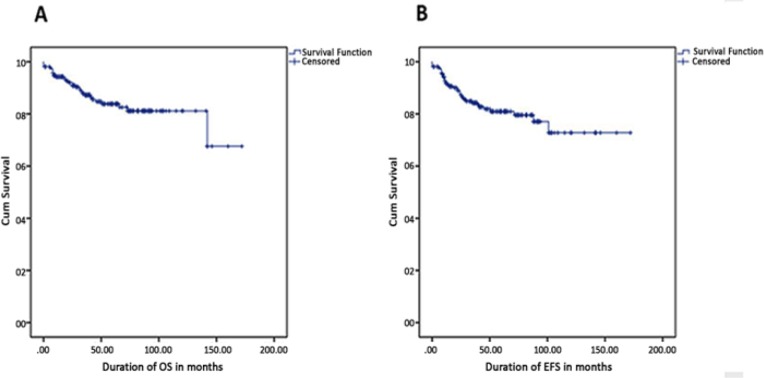
Kaplan-Meier Curves Illustrating Survival Estimates. (A), OS estimate is 85.4%; (B), EFS estimate is 82.5%

**Figure 3:**
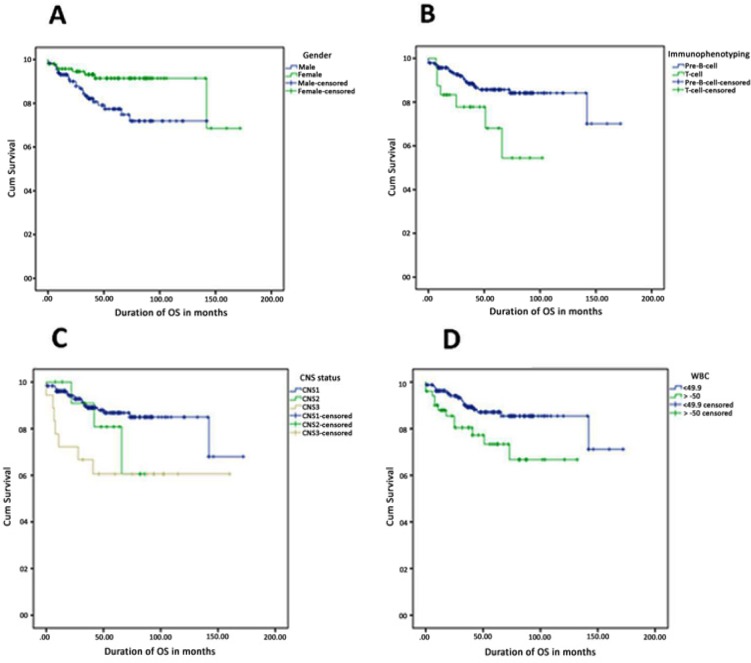
Comparison of OS for Different Risk Factors. (A), Gender: Female patients have survival advantage over male patients (91.6% vs. 80.5%, p=0.013); (B), Immunophenotype: Precursor-B-cell ALL patients have remarkably higher OS than T-cell ALL patients (87.3% vs. 70.8%, p=0.014); (C), CNS status: ALL patients with no leukemic blasts (CNS1) had highest OS followed by with CNS2 (<5 WBC/µl positive blasts) and CNS3 (≥ 5 WBC/µl positive blasts) (88.5% vs. 76.9% vs. 61.1%, p=0.014); (D),WBC count: Patient with WBC <50 had better survival than patient with WBC ≥ 50 (88.3% vs. 76.5%)

**Figure 4:**
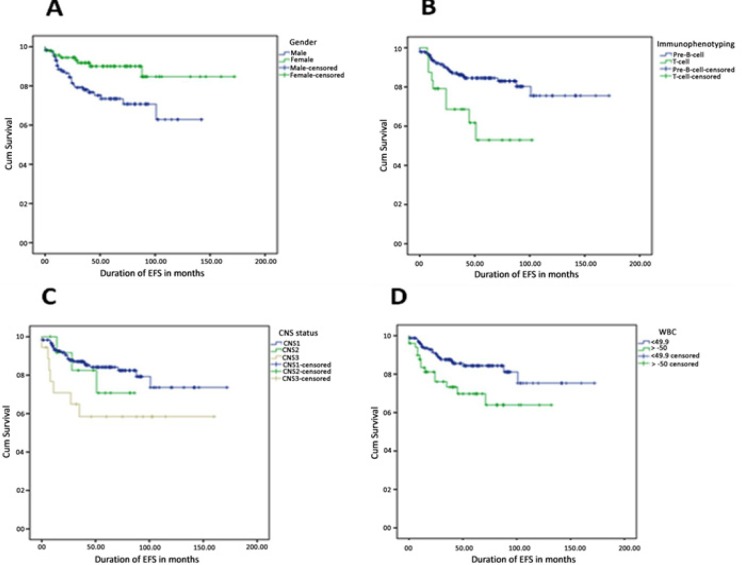
Estimation of EFS for Different Risk Factors. (A), Gender: Female patients have higher EFS than male patients (90.4% vs. 76.3%, p=0.006); (B), Immunophenotype: Precursor-B-cell ALL patients have remarkably higher EFS than T-cell ALL patients (85.1% vs. 62.5%, p=0.004); (C), CNS status: ALL patients with no leukemic blasts (CNS1) had highest EFS followed by with CNS2 (<5 WBC/µl positive blasts) and CNS3 (≥5 WBC/µl positive blasts) (85.1% vs. 76.9% vs. 61.1%, p=0.053); (D), WBC count: Patient with WBC <50 had better EFS than patient with WBC ≥ 50 (85.8% vs. 72.0%, p=0.015)

**Figure 5:**
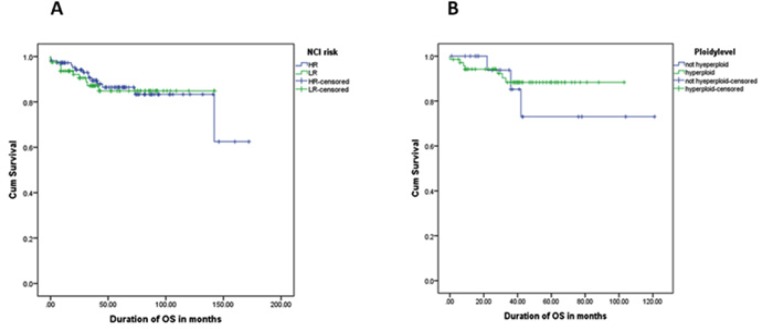
OS in pre-B ALL. (A) Precursor-B-cell ALL patients have comparable OS in HR and LR Groups (87.2% vs. 87.5%, p=0.911); (B) When stratified according to ploidy level, OS in hyperploid Pre-B ALL patients was slightly more than non-hyperploid pre-B ALL (90.1% vs. 86.4%, p=0.557)

## Results

*Patient Demographics*

Table 1 describes the study cohort in terms of demographic and clinical characteristics. Majority of the cohort (82.2%) were within the 1–9.99 years of good-risk age category except for only 3infants. Thirty-eight patients (17.8%) were of 10 years or older than 10 years. The mean age of the study cohort was 5.5 years (± 3.6). The median age was 5 years with the age range of 0.5–15 years. The cohort was consisted of 118 male (55.4%) and 95 female (44.6%) patients. According to the National Cancer Institute (NCI)/Rome risk-based stratification (Smith et al., 1996), 131 patients (61.5%) were assigned to the NCI-high-risk (NCI-HR) group, and the rest were assigned to the NCI-low-risk (NCI-LR) group. Apart from age and initial WBC count (>50×10^9 ^/L at diagnosis), immunophenotype, involvement of CNS, abnormal cytogenetics character and suboptimal response at induction were also considered during risk-group stratification.

*Clinical Characteristics*

Majority of the patients had WBC count <50×10^9^/L at diagnosis (76.1%, median = 13.5/µL with a range of 0.51–553.0/µL) with only 51 patients (23.9%) having WBC count ≥ 50×10^9^/L. Of the 213 patients, 189 (88.7%) had pre-B cell immune phenotype and 24 had T-cell immune phenotype. Eighteen patients had detectable CNS disease at diagnosis with positive leukocyte blast (≥ 5/µL; CNS 3 status), while 13 patients had positive leukocyte blasts in the CSF but without adequate pleocytosis (<5 leukocytes/µL; CNS 2 status) (Table 1).

*Cytogenetics and Molecular Genetics Study*

Many chromosome anomalies or arrangements recurrent in childhood ALL can be detected by karyotyping and fluorescence in-situ hybridization (FISH) of BM samples. Molecular genetics study, such as array-based gene expression profiling (GEP), can reveal chromosome rearrangements by evaluating levels at which the genes are expressed in samples. Karyotyping and other specific FISH were carried out on the BM samples of 144 patients. Several types of chromosomal anomalies were found. Of 138 patients with pre B-cell ALL(for whom the test was carried out), 31 patients (22%) had the most common chromosomal rearrangement, i.e., t(12, 21) translocation; of 117 patients with pre B-cell ALL (for whom the test was carried out), 39 patients (33%) had triple trisomy (4, 10, 17);of 145 patients with pre B-cell ALL (for whom the test was carried out), only 5 patients (3.4%) had MLL gene rearrangement; and 69 patients (32.4%) had other different chromosomal aberrations.

Chromosome number or ploidy is another predictor for treatment outcome. Hyperdiploidy is defined when cells contain more than 50 chromosomes (especially, if there is an extra chromosome 4, 10 or 17) or it can also be expressed as DNA index of more than 1.16. Total 133 patients were assessed for hyperdiploidy either by chromosomal karyotyping or by cellular DNA content (or DNA index). Of 133 patients, we found 28 patients with hyperdiploidy (21%): 9 patients (2 had higher DNA index too) with chromosome number >50, while 19 patients with DNA index ≥ 1.16.

*Treatment and Outcomes*

The patients were treated according to the CCG 1800 series of protocols until 2008. Thereafter, we switched to the CCG 1900 series protocols and continued until 2012. The frequency of assigning the CCG1991/IS protocol was highest among all (Figure 1A), mostly for low-risk group. Later, we again switched to COG0232 for some patients mostly in NCI high-risk group (Figure 1B). In addition, the St. Jude Total XIIIB HR and the St. Jude Total XV protocols were used to treat some patients with T-cell ALL, infantile leukemia, and acute biphenotyping leukemia. It is important to note that two patients developed treatment-related acute myeloid leukemia, and both were treated with ST JXIII/BH; they died with disease related mortality.

Table 2 presents the overall outcome estimates of the study cohort. Out of 213, 182 patients (85.4%) remained alive of which 82.6% patients had CR and only 2.8% had active disease. Thirty-one (14.6%) patients died at the end of the study either due to disease (9.9%) or due to treatment (4.7%). Four patients died during the treatment induction phase; among those four, two died of toxicity (sepsis in particular), while the other two died due to disease progression. Eleven patients (5.2%) were lost during follow-up, and 1 patient (0.5%) was referred to another center.

We obtained survival estimates of the study cohort from the Kaplan-Meier curves, which revealed the estimated OS and EFS at 5 years as 85.4% and 82.5%, respectively (Figure 2). Figure 3 compares OS for different risk factors. Female patients had better survival advantage when compared with males (91.6% vs. 80.5%; p = 0.013) (Figure 3A). Pre-B-cell ALL patients showed significantly better prognosis than T-cell All patients (87.3% vs. 70.8%; p=0.014) (Figure 3B). ALL patients with no leukocyte blast in the CSF (CNS 1) showed the highest OS followed by patients with CNS 2 (<5 leukocyte/µL) and CNS 3 (≥ 5 leukocyte/µL) status (88.5% vs. 76.9% vs.61.1%; p = 0.014) (Figure 3C). As expected, ALL patients with WBC count <50×10^9^ /L at diagnosis has higher OS than patients with WBC count ≥ 50×10^9^/L at diagnosis (88.3% vs. 76.5%) (Figure 3D). 

Estimation of EFS against different risk factors was also carried out using Log-rank (Figure 4). Like OS estimates, EFS estimates follow a similar trend for gender (female >male; p = 0.0006), immunophenotype (pre B-cell >T-cell; p = 0.004), CNS status (CNS 1 >CNS 2 >CNS 3; p = 0.053), and WBC count at diagnosis (WBC <50×10^9^ /L showed better EFS; p = 0.015). Importantly, we estimated both OS and EFS for different chemotherapy protocols used for the treatment of our study cohort and found that children’s oncology group (COG) protocol, which uses three drugs during induction, had better OS and EFS than other treatment protocols (Figures 3 and 4). 

The BM (11.7%) was primarily the site of relapse for most patients, followed by the patients with CNS status (2.3%). Testis involvement was found only in one patient (Table 1). Early response to therapy was monitored using BM evaluation on the day 14 of induction, and the result was encouraging. Out of 207 patients, who were assessed, 194 patients (91.1%) had less than 5% blast in BM (M1). Outcome at the end of the induction was even more encouraging with 205 patients (96.2%) achieving M1 status. Some of the prognostic factors, such as gender, immunophenotype, CNS status, MLL gene rearrangement, showed significant association with outcome at the end of the induction (Tables 3). The disease outcome including disease remission, death, and active disease were compared between high-risk (HR) and low-risk (LR) PreB-cell ALL patients. No significant difference in disease outcome (p=0.705) was detected between HR and LR groups. Approximately 80-85% remission, 10-15% death, and less than 5% active disease form were observed in HR and LR groups. 

Effect of ploidy level such as hyperploidy (>50 chromosomes) and non-hyperploidy on disease outcome was studied in PreB-cell ALL children with no substantial effect on disease outcome being observed. A slightly higher rate of remission was noted in hyperdiploid patients (about 90%) compared to diploid or hypodiploid patients (about 86%). In contrast, mortality rate was slightly greater in non-hyperploidy patients (around 15% died) than in the hyperploid patients (10%). However, the difference was not statistically significant (p=0.696) between hyperploid and non-hyperploid patients. 

While 90.1% patients with hyperdiploidy achieved remission, 86.4% patients, who were diploid or hypodiploid, achieved remission. Mortality was also high in pre-B-cell ALL patients with non-hyperdiploidy (71.4%). Pre B-cell ALL patients with and without chromosomal translocation t (12, 21) showed comparable outcomes (Figure 5). MRD is an important prognostic factor in ALL. In the current study, MRD was evaluated in only 51 pre B-cell ALL patients from January 2013 because of the lack of facility before 2013. Among 51 patients, 41 had negative MRD (<0.01) and 10 had positive MRD (≥ 0.01). The overall disease outcome was found to be better in negative MRD patients, as expected. Approximately 50% patients with MRD <0.01 showed remission in contrast to more than 90% patients with MRD ≥ 0.01 showing remission. This difference was statistically significant (p<0.05). Of 41 patients with negative MRD, who were in remission, the disease relapsed in 5 patients. No relapse was noted among those 10 patients with positive MRD. However, the percentage of death was higher in patients with negative MRD (about 50%) compared to the patients with positive MRD (about 5%). 

Chromosomal translocation is commonly observed in childhood ALL cases with t(12:21) being the most frequent translocation. Association of t (12:21) translocation with disease outcome in PreB-cell ALL patients was determined. Disease outcome did not vary significantly (p=0.394) between the patients with or without t(12:21) translocation. The remission rate was higher than 90% in translocation-positive patients whereas about 85% in negative patients. However, death rate was more in translocation-negative patients (around 15%) than positive patients (around 5%). The percentage of active disease was found to be similar (less than 5%) in patients belonging to both translocation positive and negative groups.

## Discussion

In an earlier retrospective study, conducted at KFSH and RC-Riyad branch, pediatric ALL patients, who received intensive therapy (CCG1800 series), had significantly improved outcome than patients who received local protocols (KFSH 81, 84, 87 and 90) (EFS at 5 years: CCG1800 series = 64.2% vs. local protocols = 30.6%; p <0.001) (Al-Nasser et al., 2008). However, despite the improvement, outcome remained lagged behind the reported EFS in North America for the same treatment protocol (64.2% vs. 75%) (Nachman et al., 1998a; Nachman et al., 1998b). Considering the gap in EFS, in the current study, we used risk-adapted different international treatment protocols to all pediatric ALL patients, treated at KFSH and RC-Jeddah during January 2002 to December 2015. The study cohort, consisting of 213 childhood ALL patients, were stratified into high-risk (HR) and low-risk (LR) groups based on the NCI/Rome criteria and were assigned risk-based treatment. The estimated OS and EFS at five years were 85.4% and 82.5%, respectively, suggesting remarkable improvement in better diagnosis, treatment practice and supportive care.

Treatment outcome not only depends on the treatment but also on different prognostic factors. Among different prognostic variables, we found significant association of gender, immunophenotype, WBC count, and CNS status with survival. Despite age being an important prognostic factor, we did not find significant association of age with outcome; however, 84% achieved remission in the “<10 year” age group (Table 3). Since elevated WBC count is a reflection of tumor burden, it is an important prognostic variable. As expected, patients with WBC count <50×10^9^ /L showed significantly better outcome.

Immunophenotype, another important prognostic factor, has been found to be associated with outcome of childhood ALL.T-cell phenotyping has been shown to have poorer outcome by CCG study group (Uckun et al., 1997). Our findings are also in agreement with the earlier studies with significantly lower remission (p = 0.016) achieved by the ALL patients with T-cell phenotyping when compared with their pre-B-cell counterpart. ALL patients with CNS disease, which have been associated with adverse prognosis (Gajjar et al., 2000; Burger et al., 2003; Pui et al., 1998), showed significantly poor outcome in our study cohort. 

Hyperdiploidy (>50 chromosomes) and chromosomal translocation (such as t (12, 21)) have been found to be associated with better prognosis (Trueworthy et al., 1992; Heerema et al., 1999; Rubnitz and Look, 1998). We evaluated only pre-B-cell ALL patients for cytogenetics and molecular genetics characteristics. As expected, patients with hyperdiploidy showed better prognosis with less mortality compared with the diploid or hypodiploid patients. Patients with hyperdiploidy are known to have better prognosis; hence, are treated as low-risk. Patients with t (12, 21) translocation and trisomy (4, 10, 17) did fairly well, and the patients with MLL gene rearrangement showed poor prognosis (p <0.01), as expected. Among the five patients with MLL gene rearrangement, who were treated using high risk protocol, two died. In addition, five patients with EZA-PBX1 [t (1;19)] were treated as HR patients of our of them on remission and alive, but the disease relapsed in one patient, who died. 

Early response to therapy has been associated with better outcome (Gaynon et al., 1997; Donadieu and Hill, 2001). BM evaluation at day 14 induction showed 91.1% patients with no blast (M1) in BM. MRD, another way to assess early response to therapy is the most prognostic factor in pre B-cell ALL and is usually performed on the BM at the end of the induction period, i.e., on the day28 or 29. Generally, patients with MRD <0.01 show better prognosis, but patients with MRD ≥ 0.01 require intense chemotherapy. In the current study, MRD was performed only on 51 pre B-cell ALL patients from January 2013, because of lack of facility before 2013.As expected, we found better disease progression and less number of deaths in 41 pre B-cell ALL patients with MRD <0.01; disease relapse was noted in only 5 out of 41 patients. However, MRD assessment of 4 of those 5 patients could be inaccurate since those were performed with moderate to severe hypocellular bone-marrow during the initial days of the facility started in our hospital. MRD evaluation of BM would have been ideal during the recovery phase.

In conclusion, this study once again proves the importance of risk-based intensive therapy with adequate supportive care in significant improvement in outcome. With the available local infrastructure, competitive survival at par with the developed countries can be achieved. Further large-scale studies with emphasis on cytogenetic and molecular genetics evaluation and MRD evaluation to assess prognosis are essential for the overall management and improved outcome of pediatric ALL patients in (KSA). 

## Data Availability

The data are available with the author and can be provided on request.

## Abbreviations

ALL: acute lymphoblastic leukemia

BM: Bone marrow

CNS: central nervous system

COG: children’s oncology group

CR: complete remission

CSF: Cerebrospinal fluid

EFS: event-free survival

EMR: electronic medical record

HR: High risk

KFSH and RC-J: King Faisal Specialist Hospital and Research Center –Jeddah branch

KSA: Kingdom of Saudi Arabia

MECCA: Middle East Childhood Cancer Alliance

MRD: minimal residual disease

OS: overall survival

SAPHOS: Saudi Arabian Pediatric Hematology/Oncology Society

SPSS: Statistical Package for Social Sciences

WBC: White blood cell
